# Predictors of Failed Outcomes in Ureteral Reconstruction: A Real-World Retrospective Study

**DOI:** 10.3390/medicina60101672

**Published:** 2024-10-11

**Authors:** Ching-Shiang Lin, Jian-Ri Lee, Kun-Yuan Chiu, Shian-Shiang Wang, Chuan-Shu Chen, Yu-Ju Lin, Chen-Cheng Che

**Affiliations:** 1 Department of Surgery, Taichung Veterans General Hospital, Taichung 40705, Taiwan; d070148@vghtc.gov.tw; 2 Department of Urology, Taichung Veterans General Hospital, Taichung 40705, Taiwan; roger@vghtc.gov.tw (J.-R.L.); kychiu@vghtc.gov.tw (K.-Y.C.); sswdoc@vghtc.gov.tw (S.-S.W.); chencs@vghtc.gov.tw (C.-S.C.); 3 Department of Family Medicine, Taichung Veterans General Hospital, Taichung 40705, Taiwan; nps798@vghtc.gov.tw; 4 Department of Medicine and Nursing, Hungkuang University, Taichung 43304, Taiwan

**Keywords:** ureter, reconstruction, radiotherapy, chronic kidney disease

## Abstract

*Background and Objectives:* Ureteral reconstruction is aimed at maintaining ureteral patency without the need for long-term catheters like ureteral stents or percutaneous nephrostomies. Different surgical strategies are adopted based on the etiology, the location of the injury, and the severity of the injury. We aimed to analyze the parameters that can predict which patients might not be free from further catheterization after reconstruction. *Materials and Methods:* This study included patients who underwent ureteral reconstruction from January 2007 to December 2021. The success of ureteral reconstruction was defined as being free from further catheterization after the operation. *Results:* A total of 184 patients underwent ureteral reconstruction. Malignant disease with ureteral invasion and iatrogenic injuries accounted for 79.9% of the cases. The majority (79.3%) did not have to undergo subsequent interventions. Predictors for a failed result of ureteral reconstruction included a history of radiotherapy (OR = 2.75, *p* = 0.01), chronic kidney disease (CKD) (OR = 3.42, *p* < 0.001), and an upper ureteric location of the injury (OR = 5.68, *p* = 0.042). *Conclusions:* A history of radiation therapy, an upper third ureteric location of the injury, and CKD were identified as predictors of a failed ureteral reconstruction. Malignant diseases, surgical methods, and repair techniques did not significantly affect the outcome of the operation.

## 1. Introduction

Reconstructing an injured ureter is challenging, particularly during emergency on-table consultation for rescue surgery. Many factors can contribute to negative clinical outcomes. Therefore, identifying these factors in advance is important for effective patient care. In many situations, such as malignancy surgery, iatrogenic ureteral injury, or stricture disease, patients may undergo ureter resection or transection [1]. The procedures for ureteral reconstruction and their outcomes can vary significantly. For urologists, ureteral reconstruction is a complex surgical procedure aimed at maintaining ureteral patency, preserving renal function, and avoiding the need for long-term catheter placement, such as percutaneous nephrostomy drainage (PCN) or double-J-stent insertion. However, various complications associated with ureteral reconstruction, including reconstruction failures, have been reported [2].

Recent reports indicate that the majority of ureteral injuries are iatrogenic, with 0.02 to 0.04% occurring due to gynecological operations and 0.24 to 5% occurring as a result of colorectal operations [3,4,5]. With the increasing use of minimally invasive procedures, the incidence of iatrogenic ureteral injuries has gradually risen [3,6]. There is a wide range of operative options for managing injured or severely stricture ureters, including laparoscopic repair, complex reconstruction, or even kidney Bench procedures. The choice depends on several factors, such as the location of the injury, the length of the defect, and the severity of the injury [7].

Efforts have been made to identify predictors of success or failure before surgery [8]. Long-term catheter placement and catheter exchange often cause considerable inconveniences in daily life, including financial problems resulting from medical expenses and an increased risk of infection and complications [9,10]. If patients do not need long-term catheter placement, this can help to avoid the above-mentioned risks and improve their quality of life. Therefore, we have defined the success of this operation as a patient who does not require further catheter placement or even a nephrectomy. We retrospectively analyzed clinical records, radiographic images, functional results, and postoperative interventions to determine the factors predicting a failed result of ureteral reconstruction.

## 2. Patients and Methods

### 2.1. Patient Population and Study Design

This study was conducted through an internal validation and retrospective assessment process and was approved by the Taichung Veterans Hospital (Taichung 40705, Taiwan) Institute Review Board (IRB No. CE22394B). From January 2007 to December 2021, patients over the age of 20 who underwent ureteral reconstruction were included. Patients who had undergone kidney transplantation or were under the age of 20 were excluded. A total of 184 patients were included. Various types of ureteral reconstruction surgeries were preformed, including the Bench procedure, ureteroneocystostomy, psoas hitch, Boari’s flap, transuretero-ureterostomy, and cutaneous ureterostomy.

### 2.2. Data Collection and Patient Follow-Up

Most patients underwent a follow-up in the outpatient department within one year postoperatively. We collected laboratory data including complete blood cell counts, differential counts, biochemical tests, serum creatinine levels, and the calculations of glomerular filtration rates using the epidemiological collaboration equation for chronic kidney disease (CKD). Serum creatinine levels were assessed preoperatively and, in most cases, postoperatively at three months and one year. We also collected imaging data including renal ultrasonography scans, intravenous urography scans, retrograde uretero-pyelograms (RUPGs), abdominal computed tomography scans, and magnetic resonance imaging data. Most patients underwent follow-ups in the outpatient department within three months postoperatively. Radiographic improvement of hydronephrosis was defined as an improvement compared to the preoperative images. Patients without radiological improvement were categorized as experiencing stagnation, progression, or even the development of new-onset hydronephrosis based on the aforementioned image examinations.

### 2.3. Criteria for Defining Success in Ureteral Reconstruction

The success of ureteral reconstruction was defined as a condition in which a patient does not require catheter placement or even nephrectomy after the operation. The decision for further intervention was typically made based on image-proven ureteral anastomotic leakage, patients’ complaints of flank pain with radiographic progression of hydronephrosis, progressive deterioration of renal function, and uremic syndrome. Clinical judgments were made by experienced clinicians based on their expertise. Subsequent interventions included PCN, prolongation of double-J-stent placement, and nephrectomy due to graft kidney infarction.

### 2.4. Statistical Analysis

Statistical analysis was performed using SAS software version 9.2 (SAS Institute, Inc., Cary, NC, USA). Continuous variables were presented as medians (interquartile ranges), and categorical variables were expressed as percentages. Laboratory data between the success and failure groups for ureteral reconstruction were analyzed using the Mann–Whitney U test. Postoperative follow-up data for both groups were individually analyzed using the Friedman test. Additionally, logistic regression with a significance level of *p* < 0.05 was used to estimate the odds ratio (OR) and 95% confidence interval (95% CI) for the relationship between variables and operation failure.

## 3. Results

### 3.1. Clinical Characteristics of the Patients

A total of 184 patients underwent ureteral reconstruction at our institution from January 2007 to December 2021. The median age of these patients was 57.6 years, ranging from 22 to 86. Among these patients, there were 62 men (34.1%) and 122 women (65.9%). A total of 35.3% of patients had hypertension (HTN), 28.3% had chronic kidney disease (CKD), and 20.7% had diabetes mellitus (DM). A single patient could have had one or more comorbidities. In addition, 43 patients (23.4%) had undergone radiotherapy for abdominal or pelvic malignancies, with 25 of them (13.5%) receiving radiotherapy after ureteral reconstruction and within one year before the end of our follow-up period. The patient characteristics are summarized in Table 1. The primary indications for ureteral reconstruction were malignant ureteral invasion and iatrogenic injuries during tumor excision (79.9%). The majority of injuries were located in the lower third of the ureter (89.1%). Additionally, 43 patients (23.4%) had received perioperative radiotherapy for their primary malignancies.

Most patients (79.3%) did not require further interventions after surgery. Additional interventions were necessary for thirty-eight patients (20.7%): thirty-one patients (16.8%) underwent percutaneous renal drainage, five patients (2.7%) had double J stents placed, and two patients (1.1%) received simple nephrectomies. The two patients who had simple nephrectomies had developed septic shock due to graft kidney infarction after the Bench procedure. A total of 59 patients (32.1%) showed radiographic improvement, defined as partial or complete relief of hydronephrosis, while 20 patients (10.9%) presented a stable status, progression, or even newly diagnosed hydronephrosis within one year of the postoperative follow-up.

### 3.2. Laboratory Data Follow-Up and Comparison of Both Groups

Laboratory data on white blood cell counts, hemoglobin (Hgb), serum creatinine, and estimated glomerular filtration rate (eGFR) for both the success and failure subgroups are shown in Table 2. We compared the success and failure subgroups’ laboratory data at three time points, namely, before the operation, 3 months after the operation, and 1 year after the operation, and analyzed whether the differences between the two subgroups reached statistical significance. Patients in the success group had significantly higher preoperative and 1-year-postoperative Hgb levels (12.10 g/dL vs. 11.10 g/dL, *p* = 0.007; 12.20 g/dL vs. 10.0 g/dL, *p* < 0.001). However, there was no statistically significant difference between the two subgroups, despite a better eGFR for the patient in the success subgroup, at any point in time.

We demonstrated the changes in the patients’ laboratory data results during the one-year-postoperative follow-up period in Table 3. In the success group, 75.5% of the patients (103 out of 146) completed the follow-up. Some cases showed temporary deterioration in eGFR levels, but these levels returned to baseline within the 1-year follow-up period. The changes in patients’ eGFR did not reach statistical significance. In the failure group, 65.7% of the patients (25 out of 38) were fully monitored, and there was a slight improvement in eGFR levels during the intervention, even though there was no statistically significant difference.

### 3.3. Predictors for the Failed Ureteral Reconstruction

Table 4 lists the predictors of failed ureteral reconstruction. In the univariate analysis, a history of radiotherapy (OR = 2.75, *p* = 0.01) and CKD (OR = 3.42, *p* < 0.001) showed significant risks. In the multivariate analysis, an upper-ureteric location of the injury (OR = 5.68, *p* = 0.042) was identified as a risk factor for reconstruction failure. Moreover, no significant differences in the outcomes were observed between the four surgical reconstruction techniques (the Bench procedure, ureteroneocystostomy with Boari’s flap surgery, transuretero-ureterostomy, and cutaneous ureterostomy), the natures of the primary lesions (malignancy, OR 2.48, *p* = 0.107), the surgical methods (laparoscopic surgery, OR 0.65, *p* = 0.511), or the postoperative radiotherapies (OR = 2.75, *p* = 0.146).

## 4. Discussion

Urinary injuries are most commonly caused by iatrogenic factors, particularly in gynecological, colorectal, and urological procedures [11]. Our study revealed that the primary indications for ureteral reconstruction were malignant urinary invasion and iatrogenic injuries during the resection of a malignancy (79.9%). The etiologies of ureteral stenosis or transection requiring ureteral reconstruction include ligation, accidental suturing of the ureter, partial or complete transection, clamping, thermal damage, or devascularization-induced ischemia [12].

To date, there is no clear and universally accepted definition of surgical success in ureteral reconstruction. Recent studies have defined success as the alleviation of hydronephrosis [13], while others have considered freedom from re-intervention as a measure of success [14]. Notably, hydronephrosis does not always improve after surgical intervention, and the extent of improvement depends on the duration and amount of urine accumulation in the renal pelvis before the operation. In many cases, the renal pelvis seldom returns to its original size and shape. Therefore, we defined the success of reconstruction as being free from further intervention, which includes PCN insertion, double J stenting, or nephrectomy. The reported success rates have varied from 77.8 to 88% in previous studies [2,14,15], figures consistent with our result (79.3%). In our study, only two patients (1.1%) required nephrectomy, a lower rate than that reported by Federico et al., who found that 4.3% of patients with unilateral renal impairment required nephrectomy [16].

The choice of treatment is multifaceted and primarily depends on the extent and location of the defects. Procedures such as the Bench procedure, ureteroneocystostomy, Boari’s flap, transuretero-ureterostomy, and cutaneous ureterostomy do not show significant differences. It is worth noting that reconstructing the upper ureter is considered more challenging than other reconstructions [8] owing to factors such as its anatomy, the limited ability to move nearby organs and tissues, and the higher anastomosis tension induced in comparison to other ureter reconstructions. Our study also produced similar results, indicating a higher risk of failure (OR = 5.68, *p* = 0.042) in upper ureter reconstructions. Therefore, techniques like ileal ureteral replacement, the ureteral Bench procedure, or buccal mucosal onlay ureteroplasty have been applied and are increasingly recommended for long upper ureter lesions [17,18].

Since most of our patients experienced ureteral injuries during gynecological and colorectal surgeries, the choice of approach often depends on the patients’ previous surgical history. Laparoscopic approaches did not increase the risk of failure, and while they showed a lower risk, the difference did not reach statistical significance (OR = 0.65, *p* = 0.511).

There have been few series reported on the recovery of renal function after ureteral reconstruction [19]. During the short-term follow-ups, the patients in the successful group experienced, on average, a slight deterioration in eGFR levels, which may have been associated with temporary tissue inflammation and partial obstruction after reconstruction. Additionally, most of our ureteral reconstructions were combined with major abdominal surgeries, which could have led to significant intravascular volume loss, hypotension, intraperitoneal injuries, and potential interference with the blood flow of the kidneys. The use of nephrotoxic drugs, such as contrast media for imaging follow-ups, nephrotoxic antibiotics, and non-steroidal anti-inflammatory drugs, can contribute to intermittent eGFR degradation [20]. On the other hand, appropriate interventions (e.g., PCN insertion, the prolongation of double-J-stent placement) for patients in the failure group improved eGFR levels in the one-year postoperative follow-up compared to their three-month follow-up value. Although no statistical differences were observed, both subgroups showed a slight enhancement in renal function during the 1-year follow-up period.

In a previous study, Wenske et al. reported progress in renal function during a two-year postoperative follow-up of patients recovering from ureteral reconstruction [13]. Khalaf et al. demonstrated that the deterioration of renal function is related to previous urinary interventions, trauma or injury, and advanced patient age, all of which have been shown to be strongly associated factors [21]. Aging is the sole factor contributing to the deterioration of kidney function over a five-year interval [22]. However, the change in eGFR levels within one to two years seems to be influenced by the reconstruction surgery employed, treatment decisions, and the management of postoperative complications.

Persistent hydronephrosis is not uncommon in patients undergoing ureteral reconstruction [23]. In our study, 20 patients (10.9%) experienced persistent hydronephrosis, which increased the risk of requiring further intervention after the operation. Although Li et al. have demonstrated that the degree of hydronephrosis can predict the return of kidney function after the release of an obstruction [24], clinical presentations and symptoms in patients do not always align completely with their imaging studies. While there may be no statistical significance in terms of increased reconstructive surgery failure in cases of malignant diseases, reconstruction failure appears to be associated with the underlying disease or its progression.

Our study identified 19 patients with radiographic improvement who still required additional interventions, and out of these, 17 (89.4%) were malignancy-related cases. When compared to patients who initially had no hydronephrosis, we should not ignore the elevated risk of needing subsequent interventions for patients who had experienced improved hydronephrosis, particularly for patients who underwent malignancy-related ureteral reconstructions. Furthermore, clinical physicians tend to be more proactive in assessing the timing of interventions for patients with CKD to prevent the progression of renal function decline to end-stage renal disease. Consequently, CKD emerged as a significant risk factor in the univariate analysis (OR = 3.42, *p* < 0.001).

Radiotherapy plays a significant role in the treatment of pelvic organ malignancies, including those that are colorectal, gynecological, urological, etc. Radiation has been reported to be a risk factor for surgical failure as shown through a comparison with non-radiated urinary reconstruction patients. Tseng et al. defined the success of surgery as the resolution of hydronephrosis, the removal of a ureter stent, and the maintenance of stable kidney function. More than half of the patients who had received radiation (10 out of 19) did not meet these strict criteria and showed a significantly higher risk compared to non-irradiated patients (OR = 3.1, *p* = 0.03) [25]. Tan et al. and Toia et al. also reported similar results, with failure rates of nearly 33% among radiation patients [26,27], which aligns with our study’s results, showing failure rates of 34.8% (15 out of 43).

As treatments continue to improve and cancer survivors live increasingly longer, urinary reconstructions are increasingly being performed in radiation fields. Pelvic radiotherapy has two main effects on urinary reconstruction. First, anastomotic healing relies heavily on the blood supply to tissues. Radiotherapy-induced microvascular damage can affect blood supply and thereby increase the risk of surgical failure. Second, radiation can lead to tissue fibrosis and bladder atrophy, resulting in impaired peristaltic function and reduced compliance and functional capacity, which may impact the success of the corresponding surgery [28,29]. Following the result of the history of radiotherapy as a predictor of failure, we analyzed whether the timing of radiotherapy played a role. In total, 58.1% of the patients (25 out of 43) with a history of radiotherapy received radiotherapy after ureteral reconstruction. Postoperative radiotherapy was associated with negative results as a failure predictor in the univariate analysis (OR 2.75, *p* = 0.146). Since tissue fibrosis caused by radiation usually occurs six months to a year after radiation therapy [30], postoperative radiotherapy has not been statistically significantly proved to be a risk factor for ureteral reconstruction failure.

In a report involving 23 patients, ileal ureter replacement achieved a 100% anatomical success rate when defined as the absence of recurrent ureteral strictures (anastomotic). However, in radiation-exposed patients undergoing ileal ureter replacement, radiation-related strictures were associated with a significantly higher rate of small-intestine obstruction and a greater need for reoperation compared to patients with other etiologies for strictures (13.0 vs. 1.2%, *p* = 0.033) [31]. Therefore, when dealing with radiation-exposed patients, careful consideration of the choice of operative repair technique is crucial.

Our retrospective study had some limitations. First, the surgeries were performed by different surgeons at our medical center, potentially introducing variations in surgical techniques and approaches. Second, our research was conducted as a single-center study with a limited sample size, which may have restricted the generalizability of the results. Further studies with larger sample sizes as well as standardized surgical and management protocols are needed to confirm and expand upon the findings of this study. We concluded that radiation therapy, reconstruction of the upper urinary tract, and CKD are predictors of ureteral reconstruction failure. Chronic kidney disease (CKD) is considered to be a patient’s underlying condition in this regard. The location of the injury and the need for radiotherapy due to cancer are also considered inevitable risk factors. Therefore, when discussing the risks of surgery with patients and their families, we must consider these three risk factors in order to minimize possible conflicts. Furthermore, postoperative follow-ups should be more proactive for these patients than for those without these risk factors. Conversely, malignant diseases, surgical methods, and repair techniques did not significantly impact the surgical outcomes. Proper medical decision for subsequent interventions can successfully maintain kidney function, even in cases where the initial reconstruction surgery has failed.

## 5. Conclusions

A history of radiation therapy, an upper-third ureter injury, and a preoperative diagnosis of chronic kidney disease (CKD) (≥stage 3) were identified as predictors of failed ureteral reconstruction. In contrast, malignant diseases, surgical methods, and repair techniques did not significantly influence the surgical outcomes. Furthermore, appropriate medical decision-making regarding subsequent interventions can successfully preserve kidney function, even in cases where the initial reconstruction surgery has failed.

## Figures and Tables

**Table 1:** Patient characteristics.

	Total (*n* = 184)	
Age, mean ± SD (years)	57.6	±13.26
Height (cm)	159.3	±8.59
Weight (kg)	60.3	±12.31
BMI, mean ± SD (kg/m^2^)	23.7	±4.07
Surgical repair technique, No. (%)		
Bench procedure	11	(6.0%)
Boari’s flap + Ureteroneocystostomy	108	(58.7%)
Transuretero-ureterostomy	53	(28.8%)
Cutaneous ureterostomy	12	(6.5%)
Surgery method, No. (%)		
Laparotomy	164	(89.1%)
Laparoscopy	20	(10.9%)
Laterality, No. (%)		
Left	92	(57.1%)
Right	69	(42.9%)
Location of injury, No. (%)		
Lower	163	(88.6%)
Middle	11	(6.0%)
Upper	10	(5.4%)
Characteristic of primary lesion, No. (%)		
Benign	37	(20.1%)
Malignancy	147	(79.9%)
Post-op intervention, No. (%)		
Without intervention	146	(79.3%)
PCN insertion	31	(16.8%)
Prolonged insertion of double-J stent	5	(2.7%)
Nephrectomy	2	(1.1%)
Radiographic improvement	59	(32.1%)
Without radiographic improvement	20	(10.9%)
History of Radiotherapy	43	(23.4%)
Postoperative Radiotherapy	25	(13.5%)
HTN	65	(35.3%)
DM	38	(20.7%)
CAD	16	(8.7%)
CVA	10	(5.4%)
Hyperlipidemia	20	(10.9%)
COPD	9	(4.9%)
Peripheral vascular disease	7	(3.8%)
CKD	52	(28.3%)
Sleep disorder	9	(4.9%)
Gout	12	(6.5%)

BMI, body mass index; PCN, percutaneous nephrostomy; HTN, hypertension; DM, diabetes mellitus; CAD, coronary artery disease; CVA, cerebrovascular accident; COPD, chronic obstructive pulmonary disease; CKD, chronic kidney disease.

**Table 2:** Comparison of laboratory data and data variation between two groups.

	Success (*n* = 146)		Fail (*n* = 38)		*p*-Value
Pre-op Cr. (mg/dL)	0.90	(0.70, 1.22)	1.02	(0.76, 1.56)	0.111
3-mo. post-op Cr.	0.89	(0.70, 1.10)	1.10	(0.70, 1.55)	0.085
1 y post-op Cr.	0.88	(0.71, 1.18)	1.17	(0.71, 2.03)	0.116
Pre-op eGFR (mL/min/1.73 m^2^)	79.60	(55.76, 100.55)	65.22	(42.41, 93.93)	0.122
3-mo. eGFR	79.45	(56.67, 101.71)	71.18	(37.91, 94.53)	0.112
1 y eGFR	79.71	(57.91, 96.17)	70.07	(36.13, 96.44)	0.203
Pre-op WBC (/µL)	7840.00	(5962.50, 10,082.50)	6610.00	(5117.50, 9512.50)	0.041 *
3-mo. WBC	6430.00	(4762.50, 8825.00)	6405.00	(4730.00, 10,025.00)	0.794
1 y WBC	6230.00	(5070.00, 8700.00)	7015.00	(6350.00, 12,775.00)	0.024 *
Pre-op Hgb (g/dL)	12.10	(10.75, 13.20)	11.10	(9.60, 12.10)	0.007 **
3-mo. Hgb	11.10	(10.00, 12.40)	10.05	(9.10, 12.38)	0.078
1 y Hgb	12.20	(10.40, 13.30)	10.00	(8.20, 11.00)	<0.001 **
Δ3-mo. post-op Cr.	−0.01	(−0.14, 0.13)	−0.01	(−0.19, 0.38)	0.765
Δ1 y post-op Cr.	0.03	(−0.10, 0.16)	0.18	(−0.13, 0.57)	0.110
Δ3-mo. eGFR	0.26	(−16.65, 13.78)	0.76	(−18.37, 23.18)	0.907
Δ1 y eGFR	−3.97	(−14.49, 10.49)	−7.37	(−26.13, 17.14)	0.408
Δ3-mo. WBC	−1095.00	(−3435.00, 505.00)	90.00	(−2807.50, 2560.00)	0.079
Δ1 y WBC	−1180.00	(−3032.50, 362.50)	780.00	(−1032.50, 6462.50)	0.001 **
Δ3-mo. Hgb	−0.65	(−1.50, 0.43)	−0.60	(−1.70, 0.50)	0.832
Δ1 y Hgb	−0.10	(−1.40, 1.00)	−1.35	(−2.55, 0.65)	0.055

Cr., creatinine; eGFR, estimated glomerular filtration rate, WBC, white blood cell count; Hgb, hemoglobin; Δ3-mo. post-op, lab data variation between three months post operation and pre-operation; Δ1 y post-op, lab data variation between one year post-operation and pre-operation. Mann–Whitney U test. * *p* < 0.05, ** *p* < 0.01.

**Table 3:** Pre-operative laboratory data and variance during follow-ups for both groups.

	*n*	Pre-Op		3-Mo. Post-Op		1 y Post-Op		*p*-Value
**Success group**								
Cr. (mg/dL)	103	0.88	(0.70, 1.29)	0.90	(0.73, 1.10)	0.91	(0.71, 1.24)	0.289
eGFR (mL/min/1.73 m^2^)	103	78.75	(55.43, 100.45)	77.59	(56.64, 94.87)	79.31	(57.30, 96.13)	0.289
WBC (/µL)	75	7830.00	(5900.00, 9530.00)	5870.00	(4690.00, 8050.00)	6010.00	(4780.00, 8700.00)	<0.001 **
Hgb (g/dL)	76	12.30	(10.68, 13.35)	11.10	(10.03, 12.38)	11.70	(10.15, 13.08)	0.024 *
**Failure group**								
Cr. (mg/dL)	25	1.01	(0.69, 1.53)	1.00	(0.74, 1.39)	1.20	(0.72, 2.05)	0.105
eGFR (mL/min/1.73 m^2^)	25	66.06	(43.66, 107.47)	71.26	(54.19, 99.03)	67.72	(32.95, 97.47)	0.105
WBC (/µL)	28	6345.00	(4525.00, 9537.50)	6405.00	(4730.00, 10,025.00)	7750.00	(6212.50, 12,925.00)	0.097
Hgb (g/dL)	28	10.95	(9.25, 11.95)	10.05	(9.00, 12.28)	9.95	(8.13, 10.95)	0.037 *

Cr., creatinine; eGFR, estimated glomerular filtration rate; WBC, white blood cell count; Hgb, hemoglobin. Friedman test. * *p* < 0.05, ** *p* < 0.01.

**Table 4:** Predictors of failed ureteral reconstruction.

Variable	Univariate			Multivariable		
	OR	95% CI	*p*-Value	OR	95% CI	*p*-Value
Age (years)	1.01	(0.98–1.04)	0.511			
Height (cm)	1.02	(0.98–1.07)	0.261			
Weight (kg)	1.01	(0.98–1.04)	0.655			
BMI (kg/m^2^)	1.00	(0.91–1.09)	0.926			
Surgical repair technique, No. (%)						
Bench procedure	Reference					
Boari’s flap + Ureteroneocystostomy	0.73	(0.14–3.69)	0.699			
Transuretero-ureterostomy	1.95	(0.38–10.04)	0.426			
Cutaneous ureterostomy	3.21	(0.47–21.80)	0.232			
Surgery method, No. (%)						
Laparotomy	Reference					
Laparoscopy	0.65	(0.18–2.35)	0.511			
Laterality, No. (%)						
Left	Reference					
Right	0.84	(0.36–1.92)	0.675			
Location of injury, No. (%)						
Lower	Reference			Reference		
Middle	1.60	(0.40–6.37)	0.507	2.14	(0.42–10.96)	0.363
Upper	2.84	(0.76–10.67)	0.123	5.68	(1.06–30.38)	0.042 *
History of radiotherapy	2.75	(1.27–5.94)	0.010 *	2.53	(0.96–6.66)	0.059
Postoperative radiotherapy	2.75	(0.70–10.75)	0.146			
Characteristic of primary lesion, No. (%)						
Benign	Reference					
Malignancy	2.48	(0.82–7.50)	0.107			
Pre-op hydronephrosis	2.08	(0.97–4.47)	0.062			
HTN	0.94	(0.44–1.99)	0.872			
DM	1.03	(0.43–2.48)	0.945			
CAD	0.24	(0.03–1.85)	0.169			
CVA	1.70	(0.42–6.92)	0.457			
Hyperlipidemia	0.96	(0.30–3.05)	0.939			
COPD	3.32	(0.85–13.02)	0.086			
Peripheral vascular disease	0.63	(0.07–5.40)	0.674			
CKD	3.42	(1.63–7.21)	<0.001 **	2.31	(0.93–5.69)	0.070
Sleep disorder	1.10	(0.22–5.54)	0.905			
Gout	3.01	(0.90–10.08)	0.074			

BMI, body mass index; HTN, hypertension; DM, diabetes mellitus; CAD, coronary artery disease; CVA, cerebrovascular accident; COPD, chronic obstructive pulmonary disease; CKD, Chronic kidney disease. Logistic regression. * *p* < 0.05, ** *p* < 0.01

## Data Availability

The data presented in this study are available upon reasonable request from the corresponding authors Che, C.-C.
